# Individualized Target Training Facilitated Transfer of Group Housed Capuchin Monkeys *(Sapajus apella*) to Test Cubicles and Discrimination of Targets on Computer Touch Screens

**DOI:** 10.3390/ani11072070

**Published:** 2021-07-12

**Authors:** Sabrina Brando, Lillian Basom, Meredith Bashaw, Caitlin Druyor, Ellen Fonte, Roger Thompson

**Affiliations:** 1 AnimalConcepts, P.O. Box 378, 03725 Teulada, Alicante, Spain; 2 Biological Foundations of Behavior Program, Franklin & Marshall College, Lancaster, PA 17604, USA; lraschi@fandm.edu (L.B.); mbashaw@fandm.edu (M.B.); caitlin.druyor@gmail.com (C.D.); erfonte1@gmail.com (E.F.); roger.thompson@fandm.edu (R.T.)

**Keywords:** capuchin monkey, positive reinforcement training, target training, learning theory, animal welfare, animal husbandry

## Abstract

**Simple Summary:**

Coercion and non-voluntary procedures can cause fear, anxiety and maladaptive behaviors for captive animals, which makes animal husbandry and care more difficult and reduces animal welfare overall. Positive reinforcement training (i.e., using rewards for desired behaviors rather than punishment for undesirable behaviors) is shown to be beneficial in reducing animals’ fear responses and in encouraging voluntary cooperation across a wide range of species. This paper outlines two experiments in positive reinforcement training to understand the importance of individualized targets in facilitating the voluntary transfer of captive capuchin monkeys (*Sapajus apella*) from their group enclosure to individual test cubicles. Experiment 1, which assigned different colored and geometric targets to each animal, rewarded animals for touching and following their assigned target into their test cubicle. The animals rapidly acquired the ability to identify their target, ultimately allowing them to cooperate by moving from one space to another voluntarily. Experiment 2 rewarded animals for spontaneously identifying their assigned target among other animals’ targets and novel targets. The animals chose their target more often than predicted by chance, although they did make some errors.

**Abstract:**

Animals in captivity often experience fear, anxiety and aggression during non-voluntary procedures, leading to adverse behaviors and ineffective outcomes for both animals and caretakers. Negative reinforcement and punishment, often due to ignorance regarding animal learning, can hurt animal welfare. However, voluntary participation through positive reinforcement training (PRT) can decrease stress related to these procedures and increase desired behaviors. Our goal was to demonstrate the positive effects of “target training” on animal welfare by training 10 captive capuchin monkeys (*Sapajus apella*) in two experiments designed to facilitate movement from a group home enclosure to a test cubicle. In Experiment 1, each monkey was assigned an individualized target (a unique shape/color combination). In daily training sessions, the animal was rewarded with a click-sounding stimulus and a food reinforcer for (a) touching the target, (b) following the respective target into a test cubicle, and (c) touching progressively smaller targets until progressing to digitized images on a computer touch screen. All 10 animals learned to approach and touch their individual physical target in one or two sessions and were able to successfully transition this behavior to an image of their target on a touch screen, although they made more errors with the touch screen. In Experiment 2, the animals were presented with other animals’ targets and novel targets. The seven animals in this experiment all touched their target at higher-than-chance rates in Trial 1 without explicit discrimination training, but only five reached the learning criteria for the task (>83% correct for three consecutive testing days. These results demonstrate that target training can make voluntary movement from group housing to test cubicles easier and benefit future animal care and procedures.

## 1. Introduction

Animals in human care must often be moved from one location to another to facilitate enclosure cleaning, animal care, and veterinary treatment. Animals that are moved involuntarily can develop fear responses, and in turn, counterproductive behaviors and stress [1]. Historically, animals were captured and/or restrained for these procedures. However, the application of animal training principles in animal care has led to beneficial changes, including a transition to the use of methods that focus on positive reinforcement training (PRT) and heightened sensitivity to the animal’s body language to avoid creating fear responses and aggressive behaviors [2].

PRT procedures give animals the opportunity to participate in experimental and husbandry procedures voluntarily, thereby mitigating the possibility of fear and/or anxiety and biological stress responses becoming associated with testing equipment, husbandry procedures, and specific personnel [2,3]. Positive reinforcement is the addition of a reinforcer to the animal’s environment following a desired behavior; PRT is the deliberate use of cues (also known as discriminative stimuli) and provision of reinforcers following desired behaviors to increase the frequency of those behaviors [4]. PRT not only promotes the desired behaviors and creates a stimulating and safe learning environment for the animals, but also affords caregivers and researchers an opportunity to build trust and a social bond with their animals [2,3]. Because the addition of the reinforcer depends on the animal’s actions, the animal has control over whether to complete the behavior and continue the training session or walk away and end the session. Studies on a range of species, including primates, demonstrate that animal welfare is improved when animals have the greater control over their physical environments and their interactions with caretakers provided by PRT programs (see [5] for review). PRT programs reduce the rates of negative cues and/or consequences for animals [2,3] and the use of negative reinforcement (see [6]). In short, PRT affords researchers the opportunity to increase the physical and psychological welfare of their animals in accordance with legal mandates and national agency guidelines [7,8].

PRT programs with both visual and auditory (e.g., vocal and/or clicker) discriminative stimuli have proven useful in many areas of animal care with captive nonhuman primates [9,10,11,12]. PRT techniques have also been used successfully to increase physical activity, provide sensory stimulation, reduce stress, and encourage compliance with basic husbandry and veterinary procedures [2]. The most common PRT technique used in animal management is target training, in which animals learn that approaching a visually distinct target stimulus results in a reinforcer which the animals desire to obtain. Marmosets (*Callithrix jacchus*), for example, have been trained to participate in their daily health care plans by following a perceptibly distinctive ‘target’ stimulus such as a plastic spoon to voluntary mount scales for weight checks [8]. Target training has also been used to shift animals into crates or to different parts of their enclosures [13]. Vocal and/or visual signals have been similarly used to shift monkeys to different enclosures [14].

Evans and colleagues [15] applied PRT through computer-based testing systems to facilitate cognitive data collection on a socially-housed group of capuchins. In this study, capuchins learned to complete cognitive tasks by moving a cursor on a computer screen by manipulating a joystick with their hands to earn reinforcers (which were dispensed automatically following a correct response). The monkeys were first habituated to the testing enclosures, then provided with food reinforcers for entering and remaining in the testing enclosures until the experimenters were able to close the doors to the main colony room without visually distressing the monkeys. The key to this effort was limiting the capuchins’ access to food for several hours prior to the training sessions and reducing distractions by installing opaque panels between the individuals. The process of training eight of their nine capuchins to enter the testing enclosure and engage in the computerized tasks took a total of 16 weeks.

The present study was prompted by the movement of the capuchin monkey colony (*Sapajus apella*) at Franklin and Marshall College (F&M) into a new facility, comprising a group-housing enclosure with adjoining individual test cubicles in October 2007. Like the facility used by Evans and colleagues [15], our new facility allowed our small group of capuchins to live in a stable social group and move into individual cubicles for short periods of noninvasive testing of their perceptual, learning and cognitive capacities. We shared Evans and colleagues’ goal [15]: to implement a PRT program in which the capuchins would move voluntarily into and out of the test cubicles and learn to respond on a touch screen to obtain reinforcers, improving their welfare by giving them more control, mitigating potentially aversive effects of physical capture and restraint [16] and facilitating individualized cognitive testing. However, we used a different training approach than Evans and colleagues [15], first training each animal to respond to a unique physical target and using a touch screen rather than a joystick. We anticipated that using unique targets for each individual would facilitate learning (particularly differentiation among targets) and using a touch screen would allow an easier transfer of the response from the physical target to the computer screen.

In Experiment 1, we studied the process of target training by employing spatially dynamic gestures (e.g., moving the hands) and vocal cues (e.g., the ‘target’) while presenting individual targets made out of laminated construction paper with a unique hue/shape combination for each monkey. We used the gesture/vocal cue/targets first to transfer our animals back and forth between the group housing enclosure and individual test cubicles and subsequently to facilitate the animals’ in responding to computer touch screens. Throughout Experiment 1, each animal was presented only with his or her own target, so the degree to which particular features of the target acquired stimulus control was not assessed. Were the animals’ ‘target’ responses controlled by a single stimulus feature of their respective digitized target (i.e., hue or shape) or, alternatively, by a compound stimulus characterized by both hue and shape? In Experiment 2, we addressed the question of single or compound stimulus control directly by testing the discriminability of each animal’s own target against a diverse set of possible alternatives.

## 2. Materials and Methods—Experiment 1

### 2.1. Ethics Statement

The training protocols were approved by the Franklin and Marshall College Institutional Animal Care and Use Committee in 2008 (2008–2009 #1).

### 2.2. Experiment 1

In Phase 1 (Stages 1–4), each animal was initially reinforced in the group housing enclosure with a click-sounding stimulus, followed by a food reinforcer for approaching and touching his or her individual target (a laminated, colored, geometric shape).

In Phase 2 (Stages 5 and 6), the animals were similarly reinforced for following his or her respective target into a test cubicle where they first generalized target-touching to differently-sized smaller targets presented behind either the mesh or glass cubicle doors (Stage 5). Finally, in Stage 6, the animals were presented with digitized targets on a computer touch screen.

### 2.3. Animals

The participants were 10 tufted capuchin monkeys (*Sapajus apella*)—three females aged 29, 10, and 7 years old, and seven males aged 13, 11, 9, 6, 5 (two animals), and 1 year old, respectively.

The animals had ad libitum access to ‘water-lickits’ and were fed daily with New World Primate Chow (Labdiet/PMI Nutrition International^TM^, St Louis, MO, USA) with a variety of fresh fruits, nuts, and vegetables. At the start of this study in 2008, all 10 animals were cared for without barriers between caretakers and animals (i.e., in free contact [16]) and were habituated to the presence of humans entering their enclosure on a daily basis with items for cleaning, caretaking, or research. They were also accustomed to the daily introduction of novel items into their living space via their enrichment program. They had participated in previous research studies that involved learning particular tasks to obtain food reinforcers (using tools to obtain honey in 1999 acquired through self-discovery, and in experiments investigating their abilities to discriminate relations between relations [17,18]) but had no experience with a formal PRT program. Capuchins are extremely ‘handsy’ and easily acclimate to touching novel items in their environment.

### 2.4. Housing and Test Cubicles

**Figure 1:**
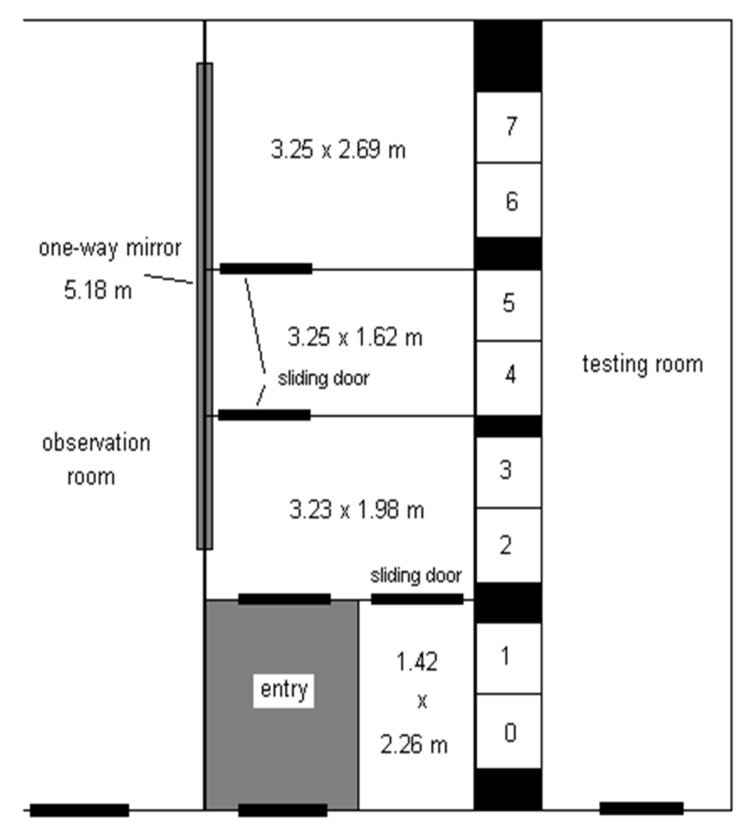
A scaled diagram of the enclosure and test cubicles.

**Figure 2:**
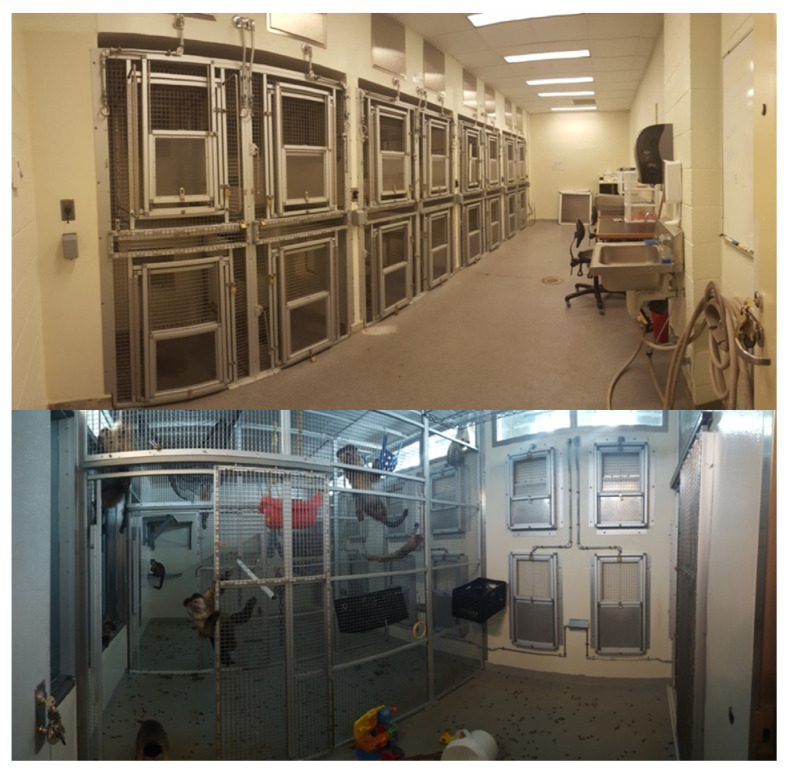
Photographs of the facility from the testing room (**top**) and the entry area (**bottom**). On the white wall on the right side of the bottom photograph, four of the 16 sliding doors to the testing cubicles are visible. These doors can be secured open or closed, so caretakers and trainers have the option to close either the mesh screen door, the solid metal panel door, or both.

The animals were housed together in the Vivarium at Franklin and Marshall College. A diagram of the enclosure is presented in Figure 1. Stainless steel mesh walls (2.54 cm sq) and sliding doors divided the overall enclosure area (24 sq. m × 2.9 m h; pictured in Figure 2) into four smaller sections. The dividing doors were normally open, giving the monkeys free range of the enclosure, except when brief separation of subgroups or individual animals was required for husbandry purposes. An observational one-way mirror (2.1 m h × 5.2 m w) extended across the length of three of the four enclosure sections, permitting unobtrusive monitoring of the animals’ behavior via either direct observation or secure live streaming using a webcam (Canon VB-C10, Huntington, NY, USA).

**Figure 3:**
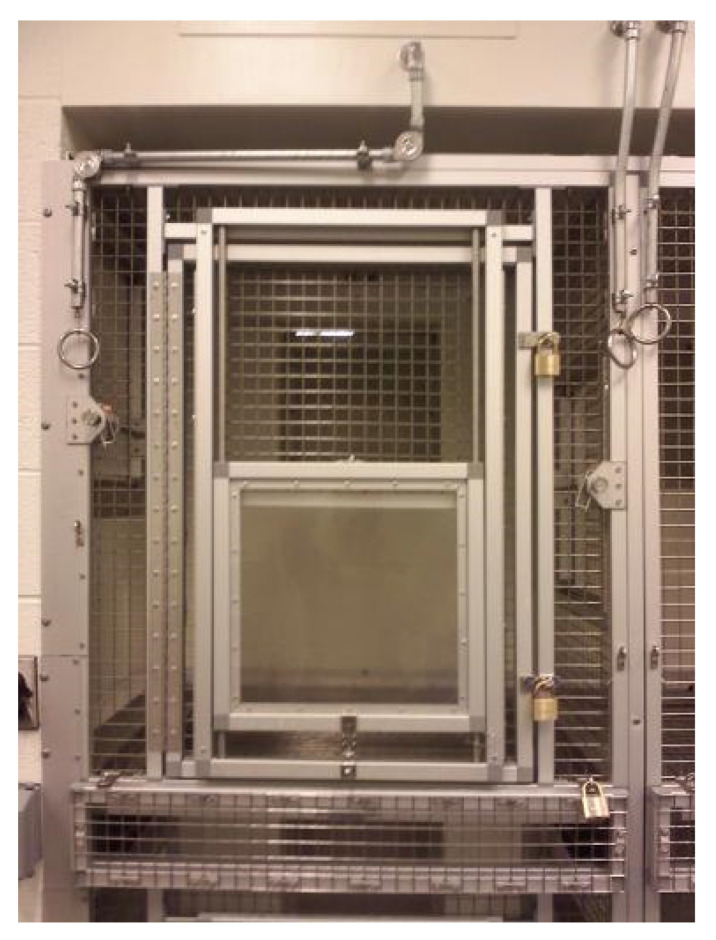
Photograph of one of the eight upper-level cubicles used in this experiment taken from the testing room. At the rear of the cubicle, the solid guillotine door leading to the capuchin enclosure is closed. At the front of the cubicle, the cubicle door consists of a stainless-steel mesh upper portion and a Plexiglas^TM^ window that can be secured at several different heights.

On the opposite solid sidewall of the enclosure were 8 pairs (two pairs per section—one upper and one lower) of guillotine doors (each 100 cm high × 51 cm wide; Figure 2 bottom). Each door allowed a capuchin to move between the enclosure and one of 16 test cubicles arranged along two rows (top and bottom) of eight in the adjoining testing room (Figure 2 top). All training and testing within the cubicles took place in the upper cubicle level. Each cubicle was 84 cm deep × 99 cm tall and 87 cm wide. The cubicle door (58.4 cm W × 89 cm L) between the cubicle and the testing room consisted of an upper portion (43.2 cm W × 35.6 cm L) made of 2.54 cm sq. stainless steel mesh and a lower Plexiglas TM window (36.8 cm square) that could be raised and locked in place at various heights as needed in testing (Figure 3).

### 2.5. Materials and Equipment

**Figure 4:**
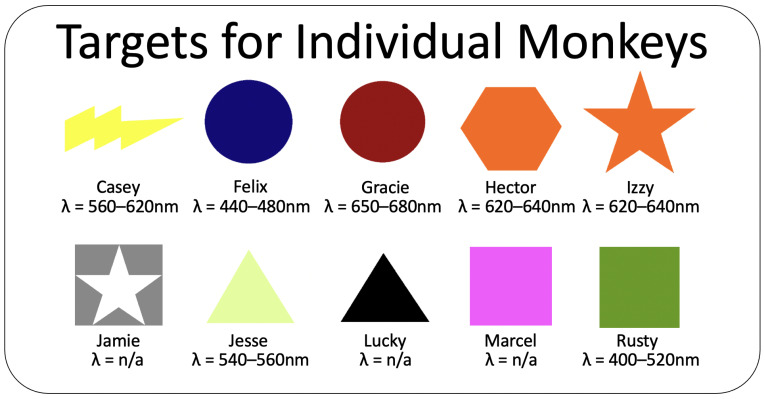
Images of the physical targets assigned to each monkey in the colony, together with the wavelengths of the light they reflected in the visible spectrum. The targets for Jamie, Lucky, and Marcel do not reflect light from a single well-defined band. Targets were originally made out of laminated construction paper and were replicated as precisely as possible when moved to the computer touch screen use. Note the overlap of shape and color for several of the targets.

Each animal’s target consisted of a colored geometric shape (9 × 9 cm) made from laminated construction paper (Figure 4). In four cases, the shape of an animal’s target was the same as that of another animal but different in color. In one case, the color of an animal’s target was the same as that of another animal, but different in shape.

In Stages 1 and 2 of Phase 1, the experimenters presented the targets by hand to the animals as described in the Methods section. In Stages 3 and 4 of Phase 1, the targets were attached to the end of a white plastic PVC pole (1.35 m in length) by means of plastic zip-ties, presented as described below. In Stage 5 of Phase 2, each animal’s target also varied in size as described below. Primary reinforcers consisted of either craisins (Oceanspray™, Markham, WA, USA) or mealworms (ZooMed™, San Luis Obispo, CA, USA). A generic dog-training clicker was used as a secondary reinforcer to bridge the delay between correct responses and the delivery of the primary reinforcer.

In Stage 6 of Phase 2 training, each animal’s target was converted into a digital image and transposed into a 200 × 200-pixel square area using a Graphic Converter. During test sessions, these images were projected onto two 38-cm-diagonal touch screens (Elo™ Touch systems ETI537L, Milpitas, CA, USA) controlled by a Mac mini™ computer (Apple, Cupertino, CA, USA) mounted on a cart. A Minolta^TM^ Luminance Meter LS-100 (Minolta, Tokyo, Japan) was used to equate the luminance levels of the digitized targets (candela per meter squared—(cd/m^2^)).

The window on the cubicle door (between the cubicle and the testing room) was raised during test sessions, and a Plexiglas™ grid template measuring 28 cm × 31 cm was affixed to the open cubicle door. An array of nine (3 × 3) rectangular holes (7.6 cm × 6.4 cm) that spatially matched nine positions on the computer screen were used to project a digitized target image. Black foam-board blinds attached to the front of the cubicles at the computer stations prevented each animal from seeing anything other than the touch screen.

In Phase 2, a computer generated ‘click’ was substituted for the hand-held clicker used in Phase 1. An automatic pellet dispenser (Med Associates^TM^ ENV-70, Fairfax, VT, USA) mounted on the cart dispensed the primary reinforcer—a single 190-mg pellet (Fruit Crunchies–Bio-serve^TM^, Flemington, NJ, USA) into a plastic dish inside the cubicle via a 2.54-cm clear plastic tube.

### 2.6. Introduction to New Facilities

New husbandry standard operating procedures (SOPs) were prompted by the capuchin colony’s transfer into new facilities in the Life Sciences and Philosophy Building’s vivarium. The animals were first collectively desensitized to their new housing by leaving the sliding doors within the group enclosure open, as well as the doors leading into and between the individual test cubicles. This allowed the animals to freely explore their new environment.

The PRT sessions described in this manuscript began approximately 10 months after their arrival in the new facility. During the 10-month period before the study, the capuchins were desensitized to entering the cubicles and hand-feeding by leaving the doors between the housing area and the cubicles open and providing some food in the cubicle. Animals who were still hesitant to enter the cubicles and/or approach humans for hand feeding were provided small food items using a cup on a long stick until they eventually came into the cubicle for hand feeding. Trainers then desensitized the animals to the opening and closing of enclosure and cubicle doors using the verbal cue “Open” each time a door was opened.

During the target training reported here, animals were often trained as a group with up to 6 trainers receiving animals as the doors opened between the group housing and the testing cubicles. Although we tracked whether a particular trainer/animal pair had an unproductive session and avoided such pairings in future sessions, animals were not paired with a consistent trainer and new trainers were introduced when the semester changed. Animals could station next to each other while learning to touch the target, shift over to another cubicle, remain in place on target while another animal shifted location, or learn target discriminations. Multiple sessions were carried out per day, ranging from 2 to 10 min. The testing cubicles were often open, allowing for interaction between animals and the caretakers and trainers. At the same time as the PRT procedures for target training, caretakers were also using PRT to train capuchins to move between adjoining cubicles in response to simultaneous vocal (“over”) and visual (dynamic pointing) discriminative stimuli.

### 2.7. Phase 1 (Stages 1–4): Acquisition of Target-Oriented Behavior

**Figure 5:**
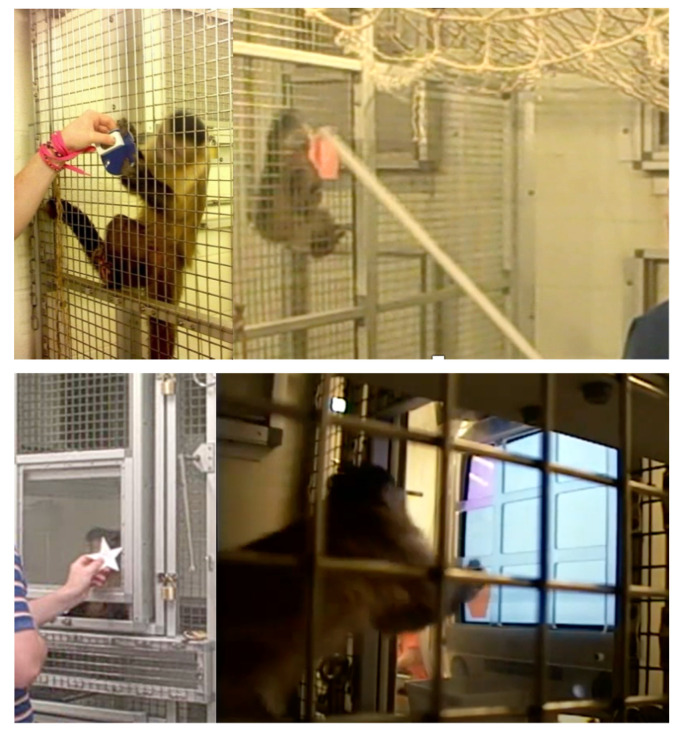
Capuchins completing the target behavior through mesh (**top left**), using the pole target (**top right**), across the Plexiglas^TM^ (**bottom left**), and on the touch screen (**bottom right**).

Phase 1 of target-oriented training consisted of four stages in which the correct response varied as described below. At each stage, an experimenter physically isolated an animal from the others in one section of the group’s enclosure via vocal and visual cues as described above. In an adjacent section of the enclosure, a second experimenter presented the individual’s physical target on the mesh wall while uttering the verbal cue, “Target” (see Figure 5, left). The correct response varied across the four training stages of Phase 1, as described below. If a correct response occurred within 5 s of a target being presented, it was immediately removed, and the animal was reinforced with the click bridge stimulus and a primary food reward. A new trial commenced 15 s later. If the animal failed to respond within the 5-s presentation period, the target was removed, and a new trial commenced following a variable 15- to 30-s time-out (TO).

#### 2.7.1. Phase 1—Stage 1: Target-Touch

On each trial, the animal was reinforced (clicker/food) for touching the target held in an experimenter’s hand at any one of a number of quasi-random positions on the mesh divider. Upon completing two successive errorless 10-trial blocks, an animal advanced to Stage 2.

#### 2.7.2. Phase 1—Stage 2: Target-Touch and Follow

Each trial consisted of ten steps. The target was first presented on the mesh divider nearest to the enclosure wall in which the one-way mirror was mounted and then moved across 10 discrete locations to the opposing wall, where the guillotine doors leading into the test cubicles were mounted. At each location a target touch was reinforced as described above. However, if the animal failed to touch the target at any given location, the target was presented again at the immediately preceding location for up to two correction attempts. An animal advanced to Stage 3 following two consecutive errorless 10-trial blocks.

#### 2.7.3. Phase 1—Stage 3: Touch and Follow Pole-mounted Target

The 10-step process within each trial was the same as in Stage 2 except that the target was now attached via a zip-tie to the end of the PVC TM pole (hereafter a pole target, see Figure 5 top right). The pole target allowed the trainer to ask the animal to move away from the mesh separating them and to respond at different heights, to station 2 animals at the same time, and to keep a distance from the animal as they were learning the task. As previously, each target touch was reinforced, and the animal advanced to Stage 4 following two consecutive errorless 10-trial blocks.

#### 2.7.4. Phase 1—Stage 4: Entry into Test Cubicle

The first experimenter reinforced an individual animal’s initial responses to the pole target with both the clicker and a food reward until touching the pole target was established. We then shaped the behavior of following the pole target into the testing cubicle by moving the target across the enclosure and into the cubicle as follows. The animal made initial contact with the pole target and was reinforced with both the clicker and food. Then the target was moved a few feet in the direction of the opposite wall eight times. As the animal approached the target at each of the eight intermediate locations, the experimenter briefly used the clicker as a ‘keep going signal’ (KGS) to indicate the monkey should keep following the target. Once the pole target reached the guillotine door on the opposite wall and the animal touched it, the first experimenter reinforced with both the clicker and food, removed the pole target, and cued a second experimenter to raise the guillotine door and present a duplicate pole target inside the testing cubicle. If the animal entered the cubicle and touched the duplicate target, the second experimenter reinforced that behavior with the clicker and food and moved the target to a new location within the cubicle. Following and touching the target one more time earned the animal a final clicker/food reinforcement, all without the use of the KGS. Hence, a total of 12 consecutive steps were required to complete a trial. Following two consecutive errorless sequences of all 12 steps, an animal advanced to Phase 2.

### 2.8. Phase 2: (Stages 5 and 6) Target Response Generalization within a Cubicle

In Phase 2, the hand-held target and the pole target were considered interchangeable, and whichever was most convenient for a given training session was used to transfer the targeting behavior to targets of different sizes, targets behind Plexiglas^TM^, and targets shown on the touch screen computer.

The animals were reinforced for responding to different-sized examples of their respective target that an experimenter manually placed on first the mesh and then the Plexiglas^TM^ portions of the test cubicle door. Animals did not easily transfer from touching the target itself touching the Plexiglas^TM^ between them and the target. Initially, the monkeys tried to reach through the mesh beside the Plexiglas^TM^ window to touch the target itself. It appeared the monkeys perceived a difference between reaching their fingers through the mesh to grab the paper target (Figure 5 top) and touching their hand to the flat Plexiglas^TM^ surface where the target was visible on the other side (Figure 5 bottom). In Stage 6, the animal’s target was presented on a computer touch screen. In both Stages 5 and 6, correct responses emitted within five seconds of target presentation were reinforced with both the click bridge stimulus and a primary reinforcer, followed by a 5-s inter-trial interval (ITI). A variable 15- to 30-s TO followed incorrect responses.

#### 2.8.1. Phase 2—Stage 5: Response Generalization to Differently Sized Targets Presented Behind Mesh or Plexiglas

Targets of various sizes (100%, 75%, 50%, and 25% of their original surface area) were presented to the animals using a performance-determined titration procedure. Over two 10-trial blocks within each 20-trial session, an experimenter manually presented a target on the PlexiglasTM or mesh portion of the cubicle door in an alternating order (e.g., MPPM). Each session began with the 100% sized target that was then titrated down from one size to the next after two successive correct trials with a given size. If the animal did not respond within 5 s, the target was increased in size by one step on the subsequent trial following a 15- to 30-s TO.

Once an animal successfully completed four trials of alternating mesh and Plexiglas trials with the 25% sized target, the experimenter presented the target on only the Plexiglas portion of the cubicle door for the following 10-trial block. Once an animal met the criterion of 80% or greater correct initial responses over two consecutive sessions of two 10-trial blocks (i.e., 40 trials in total), then it advanced to Stage 6.

#### 2.8.2. Phase 2—Stage 6: Response Generalization to Targets on Computer Touch Screen

The monkeys were first desensitized to and reinforced for targeting with the Plexiglas^TM^ template grid attached to the cubicle door backed by a clear plastic sheet. A computer touch screen on a cart was stationed approximately 25 cm back from the cubicle. Over a single 27-trial session, the experimenter presented each animal’s physical target by hand at one of the nine (3 × 3) grid locations in a quasi-random order. If the animal responded to the target within 5 s, it was reinforced as described previously, followed by a 5-s ITI. Failing to respond within 5 s, or responding to any other grid location, resulted in a variable 15- to 30-s TO.

Following the initial 27-trial active desensitization session, the plastic sheet backing the 3 × 3 Plexiglas^TM^ grid was removed and the touch screen on the computer cart was placed flush against the grid. On each subsequent 27-trial session, a digitized target (200 × 200 pixels) appeared on the touch screen at one of the 9 grid locations in a quasi-random order and remained there for a maximum of 15 s. Hence, over 27 trials the animal’s target appeared at each grid position three times. If the animal touched its target within 15 s, the animal was reinforced by a computer-generated click bridge, followed immediately by a primary reward delivered manually through a small, human finger-sized 8-cm circular opening in the black foam board blind immediately to the left of the touch screen.

If the animal touched an incorrect grid position or failed to respond within 15 s to any grid position, the trial was terminated and followed by a 5-s ITI over the first five 27-trial sessions, as well as a 3-s ITI on any subsequent session. Following an initial-trial error, the digitized target appeared in the same grid position for up to three correction trials. If the monkey touched the target on a correction trial, it was reinforced, and the next computer-programmed trial was presented after the ITI. This was also the case if the animal failed to respond correctly after three correction trials.

## 3. Results—Experiment 1

### 3.1. Phase 1: (Stages One to Four) Target Training with Physical Targets

**Figure 6:**
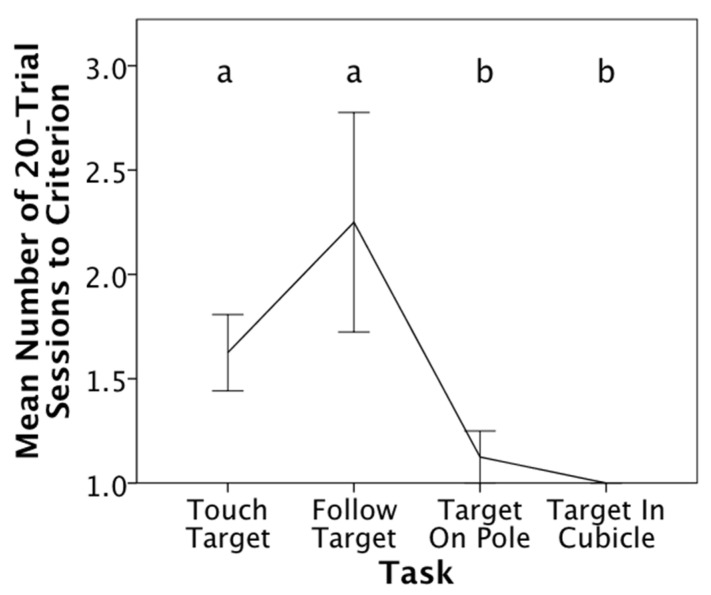
Mean Number of 20-trial sessions taken to reach the criterion of two consecutive errorless 10-trial blocks for each of the four training stages in Phase 1. Errorless responding would result in reaching the criterion in the initial session. Different letters represent significant differences in mean sessions to learn the task; error bars show standard errors.

Overall, the monkeys took significantly more sessions to reach the passing criteria in Stages 1 and 2 than in Stages 3 and 4. In Stage 1 (touch hand-held target), three of the ten animals met the two consecutive, errorless 10-trial blocks criterion within the initial 20-trial session. The remaining seven animals met the criterion within two sessions (all animals M_Touch_ = 1.7 sessions). Similarly, when learning to follow and touch the hand-held target (Stage 2), four animals responded without error and reached the performance criterion in the initial 20-trial session. However, only one of these four animals was similarly errorless in Stage 1. The remaining six animals met the Stage 2 criterion within two and five 20-trial sessions (all animals M_Follow_ = 2.1 sessions; excluding errorless animals M_Follow_ = 2.8 sessions). Only one animal made an error in Stage 3 (follow and touch pole target) M_Pole_ of 1.1 sessions) and all the animals were errorless in Stage 4 (follow and touch target in cubicle). Hence, all animals reached this criterion in fewer trials than in Stages 1 and 2, F(1,3) = 8.9, *p* = 0.05, partial Eta^2^ = 0.41 (Figure 6).

### 3.2. Phase 2: (Stage 5). Response Generalization to Reduced-Sized Targets Presented Behind Mesh or PlexiglasTM Cubicle Doors

Nine of the ten animals were errorless in reaching the performance criterion in the minimum possible two sessions when their respective individual target was systematically reduced in size, regardless of the barrier (mesh or Plexiglas^TM^) in use. The remaining animal (Lucky) responded without error when her smallest target was presented behind the mesh barrier. However, her performance decreased when it was presented behind the Plexiglas^TM^ barrier, making three such errors (i.e., failing to respond within 5-s) in Session 1, as well as one error in Session 3. Nevertheless, she reached the criterion in an additional five 20-trial sessions.

### 3.3. Phase 2: (Stage 6). Response Generalization to Reduced-Sized Targets Reduced in Size and Presented On Grid and Computer Touch Screen

All animals responded within the requisite 5-s target presentation and successfully generalized, i.e., transferred, the target behavior from the physical target to the digitized targets. Their performances in relation to digitized targets presented on a touch screen presented an average of 5.6 27-trial sessions in two weeks. However, requiring the animals to first touch the physical target’s location on the grid and then touch the digitized images on the touch screen proved to be more challenging, as reflected in the low but consistent number of errors. We calculated the error rate for each session by dividing the number of errors in that session by the 27 trials in each session.

**Figure 7:**
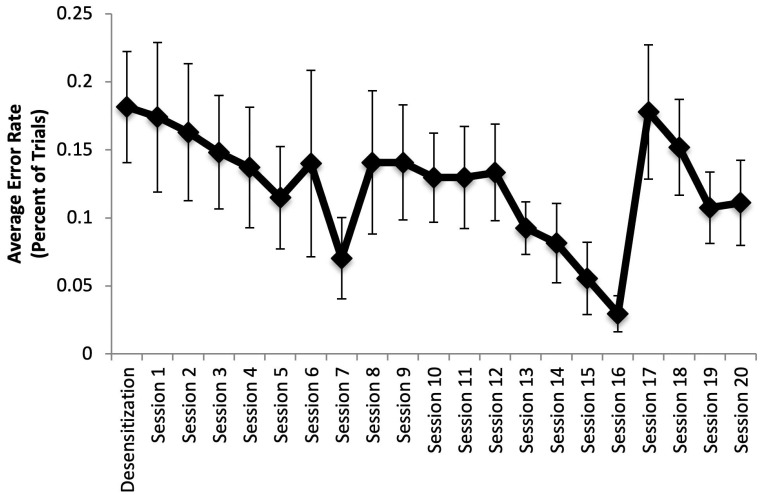
Response error rate by session in Phase 2: Stage 6, averaged across capuchins. Error rate was calculated by determining the number of errors divided by 27 trials per session. No significant differences were found across sessions.

On average, the animals made errors on 18 percent of the 27 trials in the initial desensitization session with the grid and on 12 percent of 27 trials per session when their respective targets were presented on the touch screen. However, there was no significant change in the error rate across sessions, F(4.4,35) = 1.48, *p* = 0.23, Eta^2^ = 0.16 (Figure 7). Moreover, the number of errors was not significantly affected by where the target appeared within the 3 × 3 touch screen grid, F(8,72) = 0.71, *p* = 0.69, Eta^2^ = 0.07.

## 4. Discussion—Experiment 1

Overall, the results indicate that for all animals, the location, size, and manner of presentation of their respective target were not necessarily distinguishing characteristics of the task. Once an animal learned to touch its respective target, regardless of where it was presented in Phase 1 (Stages 1–2), all but one animal touched the target presented on the pole (Stage 3), and all ten animals were similarly errorless in following the pole target from the housing area into a cubicle (Stage 4). Similarly, only one had difficulty in generalizing the response across target sizes and recognizing the target through different barriers (Stages 5 and 6). Three of the four total errors occurred in the first session. The difference in learning between Stages 1 and 2, in which animals needed more sessions to acquire the requested task, compared to Stages 3 and 4, likely reflects that the initial training to touch the target was perceived by the capuchins as a new task, whereas generalizing from the hand-held target to the pole target and following the target into the cubicle was simply an extension of the task they had already learned. That is, once the animals had acquired stage 2 of following the target, it appeared to be quite easy for the animals to follow the target on the pole and also into the cubicle. The individual differences we observed could be due to the animal’s personality, age, and experiences, or simply reflect that animals learn at different rates [19]. Ultimately, all animals learned the tasks. The open-door policy we used in order to avoid making animals feel trapped, the short and frequent training sessions, and the ability to see others during training (allowing for social learning) likely all contributed to the rapid acquisition of the different behaviors.

Could the increased error rate with the digitized targets be related to the absence of any extraneous cues previously associated with the experimenters? We think it unlikely, given the number of individuals testing the animals throughout all phases and stages of training. Instead, we note that in all the training stages preceding that with the digitized targets, an error was recorded only if an animal failed to touch the target within the specified time interval. However, the digitized task also recorded an error if the animals touched a section of the grid where the target did not appear or attempted to respond with both hands. The most common errors observed in the transition to the digitized targets were individuals ‘resting’ their hands on another part of the screen after a trial was initiated (often while they ate their reinforcement from the previous trial), touching the target with both hands, or touching an area of the screen that contained neither a target nor a distractor. As the equipment stayed in place, and animals could freely move, including leaning as they wanted, it is not surprising to see the types of errors they made. Although we cannot rule out increased errors resulting from the absence of experimenter cues, we believe the increased error rates associated with the digitized targets were more likely a consequence of this broader definition of an error. We recommend that future studies should consider using a ‘get ready signal’ to cue the animal that a trial is about to begin. Such a signal would cue the animal to stop touching the screen, finish eating, and attend to the screen prior to the start of the next trial, which could reduce the errors we most commonly observed. Alternatively, inserting an already-learned task (in our case, simple target touch trials) between discrimination task trials might also reduce errors in the discrimination task by increasing the capuchin’s overall success with the touch screen and therefore reinforcement rates.

## 5. Materials and Methods—Experiment 2

### 5.1. Experiment 2: Target Discrimination

In Experiment 1, each animal was exposed only to its individual target, leaving open the question of whether or not one stimulus dimension (shape or color) was more salient than the other. If not, and assuming the absence of a preference for novelty, then an animal should continue to choose her or his own target even when paired with that of another animal in which neither stimulus dimension matches his or her own. Alternatively, higher error rates might be anticipated if one or both of an animal’s stimulus dimensions overlaps with those of another animal. An analysis of error rates could then be evidence of a possible overshadowing stimulus control.

Alternatively, absent any evidence of discriminative control by a target’s specific features, an animal might well choose randomly, regardless of whether or not its target did or did not overlap with the incorrect alternative. The aim of Experiment 2 was to test these two possibilities.

### 5.2. Animals

Seven of the ten capuchins completed Experiment 2. Of the three that did not, one female had died, and another female was preoccupied with caring for her new-born infant. One male developed a habit of touching the touch screen targets with his mouth or head rather than his hand, leading to inconsistent recording of his responses by the computer program. Experiment 2 began approximately 14 months after the animals were moved to the new facility.

### 5.3. Equipment

Test sessions were conducted in either one of two test cubicles (at opposite ends of the cubicle row) to which the two Plexiglas grid templates (28 cm × 31 cm) and 38-cm-diagonal touch screens (Elo Touch SystemsTM ETI537L, Milpitas, CA, USA) were respectively affixed.

### 5.4. Stimuli

A total of 54 target stimuli were used, including the ten used in Experiment 1. The luminance of the nine colors (black, white, dark green, light green, blue, orange, yellow, red and pink) were measured with a spectrometer (Ocean Optics^TM^ model USB4000-VIS-NIR, 350–1000 Nm, Dunedin, FL, USA) and replicated in the touch screen format. The remaining 44 new ‘distractor’ target stimuli were created such that all six shapes and nine colors were combined, controlling for luminance (see Table 1), creating a total of 53 unique trials per session in which an animal’s target (S+) was paired with each other target. These pairings were divided into two categories: pairings with an S− that did not overlap the S+ in either color or shape and pairings where an S− overlapped with the S+ in either color or shape.

### 5.5. Procedures

On each trial, two targets (S+ and S−) were presented quasi-randomly on the touch screen at two of the nine grid positions delineated by the attached Plexiglas tm template. Within each 53-trial session, a subject’s training target (S+) was paired with one of the three types of possible S− stimuli described above. If the animal touched his or her target (S+) within 15 s she or he was reinforced with a computer generated ‘click’ bridge, followed immediately with a primary reward. However, if the animal touched the incorrect target (S−) the trial was terminated and followed by a 5-s ITI over the first five sessions and 3 s thereafter, given the animals’ promptness in engaging the task across the sessions.

## 6. Results—Experiment 2

In this experiment, the animals were required to discriminate their respective training targets used in experiment one from the other 53 possible shape/color target combinations by touching the former when a pair of stimuli appeared on the touch screen. On the initial trial, the monkeys correctly identified their respective targets (M = 77% correct, range = 65%–89% correct) significantly more often than would be predicted by chance (50% correct; t(6) = 7.45, *p* < 0.001). Nevertheless, their overall Trial-1 success was influenced by similarities between their target and the S− distractor.

**Figure 8:**
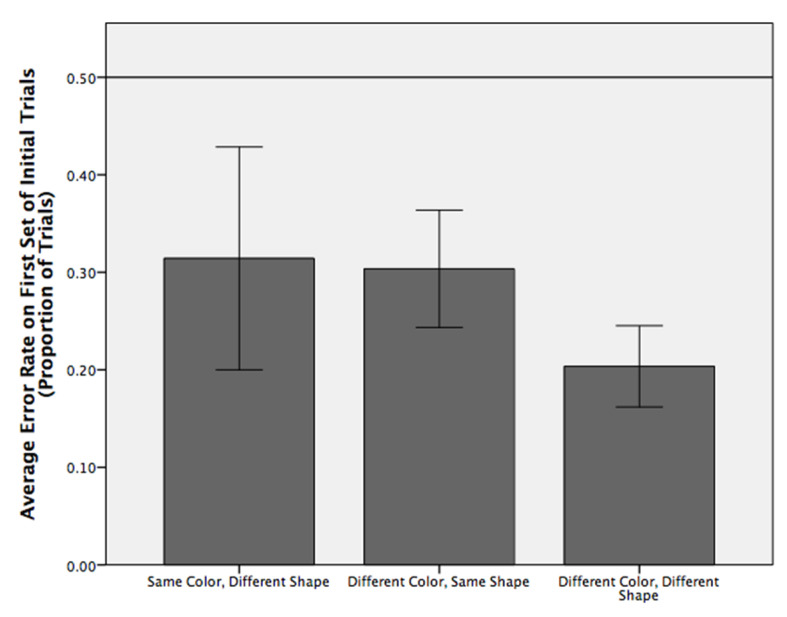
Error rate on the first trial for different trial types. No significant difference was found between conditions (F(2, 12) = 0.68, *p* = 0.53, partial Eta^2^ = 0.10). Error rate was calculated by determining the number of errors divided by the number of trials of that type presented in the 53-trial session. Reference line shows chance performance; error bars show +/− 1 SE. Performance was significantly better than chance in the “different color, same shape” and the “different color, different shape” conditions, but not in the “same color, different shape” condition.

Trial-1 performance was significantly above chance when a target was paired with a S− distractor differing in both color and shape (*n* = 40 pairs, t(6) = 7.11, *p* < 0.001, leaving open the question as to whether one dimension was more salient (i.e., overshadowed) than the other. Trial-1 performance was significantly above chance if an S− distractor differed from the S+ target in color but not shape (*n* = 8 pairs, t(6) = 3.27, *p* = 0.017). However, correct Trial-1 performance was not significantly above chance if the S− distractor was of the same color as the S+ but differed in shape (*n* = 5 pairs, t(6) = 1.63, *p* = 0.155). This dichotomy in results indicates that the capuchins attended primarily to color and not shape to identify their respective targets. Interestingly however, mean error rates were similar in cases where the target and distractor were not only the same color, but also when the target and distractor were the same shape. Nevertheless, the variability among individuals was much higher when both the target and distractor were the same color (Figure 8), suggesting that those individuals were using color to identify their target (and perhaps therefore initially had more difficulty discriminating their target from a same-color distractor), whereas other animals were not using color and made this discrimination more easily.

**Figure 9:**
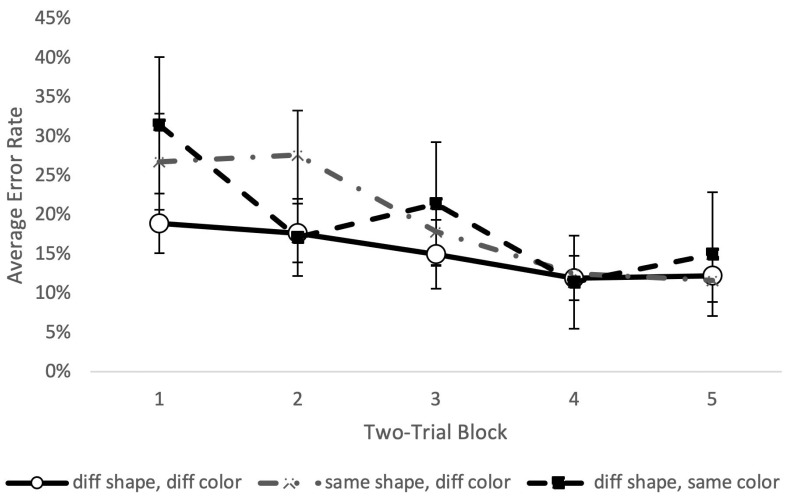
Mean error rate decreased significantly across trial blocks. Error rate was calculated by determining the number of errors divided by the number of trials of that type presented in the 53-trial session. There was no significant effect of trial type (F(2,12) = 1.22, *p* = 0.33, partial Eta^2^ = 0.16).

Despite starting out with better-than-chance performances that significantly improved across trials (Figure 9), five of the seven monkeys reached the learning criterion (better than 83% correct on three consecutive testing days) over 12 days of testing. Two monkeys met the criterion as soon as possible (on Day 3), two took seven days of testing, and one took 10 days. The failure of the two remaining monkeys to meet the performance criterion cannot be explained by poorer initial performances. Neither monkey had more errors on Day 1 than those monkeys that met the criterion (t(5) = 0.07, *p* = 0.95).

## 7. Discussion—Experiment 2

Experiment 2 revealed that all the animals discriminated their targets from those of the other animals despite never having been explicitly trained to do so. Colors were randomly assigned to the shapes, and it could be that the color in combination with a shape influenced the strategy that the animals were using, discriminating on the basis of shape or color. Individual monkeys that were using color to identify their target (and perhaps therefore initially had more difficulty discriminating their target from a same-color distractor), could potentially have performed better if the shape had been salient to them. Shapes like the ones featured in Figure 4 for Casey or Jesse or Izzy could potentially be more challenging than solid shapes like those of Felix or Rusty. Future studies could consider testing different colors and shapes with different individuals to see which factors aid in learning and which factors might increase the rate of errors.

## 8. Conclusions

In the present study, all the animals successfully generalized their performances at each stage to digitized targets presented on a touch screen in Phase 2, Stage 6, over an average of 5.6 and a maximum of twenty 27-trial sessions across two weeks of testing. These results compare more than favorably with those reported earlier by Evans and colleagues [15], who used a computer-controlled testing method to separate individuals within a socially housed colony of nine tufted capuchin monkeys and to train them to manipulate a joystick to make choices in a series of psychomotor and conceptual tasks. Evans and colleagues stressed that their method of gradual habituation was not only efficient but also relatively quick; after habituation, training their animals to individually enter, remain confined in the test enclosures, and complete computerized cognitive tasks required approximately 16 weeks. In our study, all of the animals in our study rapidly attained touch screen proficiency (i.e., Phase 2, Stage 6) in 4 weeks.

A variety of factors likely contributed to our capuchins’ faster training. Several aspects of our PRT procedures were chosen to maximize training speed: desensitization to the cubicles and hand feeding preceding PRT, the use of individual targets, more frequent and shorter training sessions (also see [20]), the use of smaller approximations during the shaping process [21,22] and the use of touch screens rather than joysticks all likely shortened our training times. In addition, social interactions among capuchins might have contributed to the shorter training times in our study in two ways. First, during Evans and colleagues’ study, their capuchins transitioned from a single group to two groups as a result of intermale aggression, whereas the animals in the present study stayed in a single social group. Social instability is a stressor for primates [23] and stress impairs cognitive function [24]. Second, in the present study capuchins had the opportunity for social learning as animals could watch each other’s training sessions, which may have especially facilitated discrimination training by providing visual access to distractors (the other monkey’s targets) even without explicit discrimination training. Together, these factors are likely to have contributed to conditions in which there was less stress and more opportunities to receive reinforcement, which would be expected to increase learning speed. We did not collect any data on personnel and/or time investment with the monkeys or possible learning through passive or active training-related activities during the initial introduction of the animals to their new housing/testing situation, which could also account for some of the differences found. Future research should disaggregate these factors to identify the most efficient training procedure for capuchins.

Once using the touch screen, the capuchins in our study were also able to successfully discriminate their target from distractors without explicit training. However, some individuals exhibited chance performance when asked to differentiate their target from a distractor that was the same color but a different shape. This suggests that some of the individuals were attending to color, rather than shape, when identifying their target. Even though the capuchins had not been asked to choose among the targets prior to Experiment 2, they were able to observe some training sessions with other individuals and may have learned how their target differed in appearance from other targets. Two animals (Hector and Izzy) used targets that were exactly the same color and others may have appeared similar because capuchins’ color perception differs from that of humans, and male capuchins may be dichromatic [25]. The color vision of the particular individuals in this study has not been assessed. Capuchins with a target that was perceived to be similar in color to another individuals’ target may have relied more on shape to identify their target, resulting in fewer errors when asked to choose between their target from same-color distractors; monkeys with a target color that was unique may have been more reliant on color to identify their target and therefore had more difficulty discriminating it from same-color distractors.

In conclusion, group housing greatly enriches the lifestyles of nonhuman primates [26,27]. When animals are not trained to voluntarily separate and/or enter transport cages and restraining chairs, the other option is to use less animal-friendly methods such as negative reinforcement or physical restraint [6,16] to catch them. Our results with target training and those of Evans and colleagues [15] demonstrate that it is possible to train animals to voluntarily enter test chambers/cubicles, thereby mitigating the stress and potential for injury to either animals or humans associated with traditional methods of separating individuals for testing. Target training procedures also have the added benefit of allowing researchers and animal caretakers to easily manipulate the order in which individual animals within a group can be moved voluntarily from one place to another.

## Figures and Tables

**Table 1:** Measurements of luminance (cd/m^2^) taken from the physical targets and the digitalized targets appearing on the touch screen for the target discrimination task to ensure that luminance levels on the computer screen and on the physical targets were comparable. The specific targets assigned to individual monkeys (Figure 2) are shaded in gray. Distractors are unshaded. Bold text indicates the average luminance for each color and shape; luminance is fairly consistent across shapes but varies widely across colors.

	Shape						
Color	Circle	Square	Triangle	Star	Hexagon	Light. Bolt	AVERAGE
Red	9.6	10	10	10.5	10.8	22	**12.15**
Pink	39	38	37	40	42.5	42	**39.75**
Orange	30	33	32	34.6	32	37	**33.1**
Yellow	101	99	93	96	105	85	**96.5**
D. Green	32	28	30.5	28	29	34	**30.25**
L. Green	88	94	105	82	91	76	**89.33**
Blue	4	4	4	4.7	4	12	**5.45**
Black	2	1.5	1.4	2.9	1.5	10.5	**3.3**
White	103	101	112	97	110	95	**103**
**AVERAGE**	**45.4**	**45.39**	**47.21**	**43.96**	**47.31**	**45.94**	**45.87**

## Data Availability

The data presented in this study are available on request from the corresponding author. The data are not publicly available because Institutional Animal Care and Use Committee approval was not obtained for data sharing at the time of the study.

## References

[1] McMillan J.L., Perlman J.E., Galvan A., Wichmann T., Bloomsmith M.A. (2014). Refining the pole-and-collar method of restraint: Emphasizing the use of positive training techniques with rhesus macaques (Macaca mulatta). J. Am. Assoc. Lab. Anim. Sci..

[2] Brando S.I. (2012). Animal learning and training: Implications for animal welfare. Vet. Clin. N. Am. Exot. Anim. Pract..

[3] Brando S.I. (2010). Advances in husbandry training in marine mammal care programs. Int. J. Comp. Psychol..

[4] Skinner B.F. (1938). The Behavior of Organisms.

[5] Allard S.M., Bashaw M.J., Kaufman A.B., Bashaw M.J., Maple T.L. (2019). Empowering zoo animals. Scientific Foundations of Zoos and Aquariums: Their Role in Conservation and Research.

[6] Perlman J.E., Bloomsmith M.A., Whittaker M.A., McMillan J.L., Minier D.E., McCowan B. (2012). Implementing positive reinforcement animal training programs at primate laboratories. Appl. Anim. Behav. Sci..

[7] EUPrim-Net. (n.d.) Positive Reinforcement Training. http://www.euprim-net.eu/network/prt.htm.

[8] National Research Council (US) (2010). Guide for the Care and Use of Laboratory Animals.

[9] Gillis T.E., Janes A.C., Kaufman M.J. (2012). Positive reinforcement training in squirrel monkeys using clicker training. Am. J. Primatol..

[10] McKinley J., Buchanan-Smith H., Bassett L., Morris K. (2003). Training common marmosets (Callithrix jacchus) to cooperate during routine laboratory procedures: Ease of training and time investment. J. Appl. Anim. Welf. Sci..

[11] Savastano G., Hanson A., McCann C. (2003). The development of an operant conditioning program for new world primates at the Bronx Zoo. J. Appl. Anim. Welf. Sci..

[12] Wagner A.R., Logan F.A., Haberlandt K. (1968). Stimulus selection in animal discrimination learning. J. Exp. Psychol..

[13] Office of Laboratory Animal Welfare, United States National Institutes of Health Report to Office of Extramural Research Acting Director on Office of Laboratory Animal Welfare (OLAW) Site Visits to Chimpanzee Facilities–July 2010. http://grants.nih.gov/grants/olaw/Report_on_OLAW_Visits_to_Chimpanzee_Facilities.pdf.

[14] Scott L., Pearce P., Fairhall S., Muggleton N., Smith J. (2003). Training nonhuman primates to cooperate with scientific procedures in applied biomedical research. J. Appl. Anim. Welf. Sci..

[15] Evans T.A., Beran M.J., Chan B., Klein E.D., Menzel C.R. (2008). An efficient computerized testing method for the capuchin monkey (Cebus apella): Adaptation of the LRC-CTS to a socially housed nonhuman primate species. Behav. Res. Methods.

[16] Laule G.E., Bloomsmith M.A., Schapiro S.J. (2003). The use of positive reinforcement training techniques to enhance the care, management, and welfare of primates in the laboratory. J. Appl. Anim. Welf. Sci..

[17] Flemming T.M., Rattermann M.J., Thompson R.K.R. (2006). Differential access to and use of reaching tools in social groups of capuchin monkeys (Cebus apella) anand human infants (Homo sapiens). Aquat. Mamm..

[18] Thompson R.K.R., Hagman C.E., Dotov D.G., Templer V.L. Can capuchin monkeys (Cebus apella), like humans discriminate relations-between-relations? Maybe…maybe not. Proceedings of the 14th International Conference on Comparative Cognition.

[19] Carere C., LoCurto C. (2011). Interaction between animal personality and animal cognition. Curr. Zoöl..

[20] Fernström A.-L., Fredlund H., Spångberg M., Westlund K. (2009). Positive reinforcement training in rhesus macaques—training progress as a result of training frequency. Am. J. Primatol..

[21] Fernandez E.J. (2020). Training petting zoo sheep to act like petting zoo sheep: An empirical evaluation of response-independent schedules and shaping with negative reinforcement. Animals.

[22] Slater C., Dymond S. (2011). Using differential reinforcement to improve equine welfare: Shaping appropriate truck loading and feet handling. Behav. Process..

[23] Capitanio J.P., Cole S.W. (2015). Social instability and immunity in rhesus monkeys: The role of the sympathetic nervous system. Philos. Trans. R. Soc. B Biol. Sci..

[24] Sapolsky R. (2005). Why Zebras Don’t Get Ulcers.

[25] Gomes Ú.R., Pessoa D., Tomaz C., Pessoa V.F. (2002). Color vision perception in the capuchin monkey (Cebus apella): A re-evaluation of procedures using Munsell papers. Behav. Brain Res..

[26] Novak M., Suomi S.J. (1991). Social interaction in nonhuman primates: An underlying theme for primate research. Lab. Anim. Sci..

[27] Baker K.C., Dettmer A.M. (2016). The well-being of laboratory non-human primates. Am. J. Primatol..

